# Beyond the Earthquake: Cardiovascular Consequences and Lessons for Colombia

**DOI:** 10.5334/gh.1594

**Published:** 2026-10-16

**Authors:** Santiago Sierra-Castillo, David Aristizabal-Colorado, H. A. Nati-Castillo, Jhan Saavedra-Torres, Juan S. Izquierdo-Condoy

**Affiliations:** 1 Department of Epidemiology, Universidad CES, Medellín, Colombia; 2 Department of Cardiology, Universidad de Antioquia, Medellín, Colombia; 3 Department of Gastroenterology, Universidad CES, Medellín, Colombia; 4 School of Health Sciences, Fundacion Universitaria Vision de las Americas, Pereira, Colombia; 5 One Health Research Group, Universidad de las Americas, Quito, Ecuador

**Keywords:** earthquakes, cardiovascular diseases, disaster medicine, health services accessibility, healthcare disparities, Colombia

## Abstract

An earthquake is an event that affects populations across multiple dimensions, extending far beyond trauma and immediate mortality. Cardiovascular events are particularly important during the acute phase and in the first days after the disaster, when physiological stress, disruption of healthcare services, medication interruption, and barriers to timely medical attention may increase cardiovascular risk. Previous evidence has documented increases in myocardial infarction, stroke, arrhythmias, cardiovascular mortality, and decompensation of chronic cardiovascular disease following major earthquakes. In Colombia, the recent earthquake affecting Chocó and neighboring regions highlights the particular vulnerability of populations facing geographic isolation and unequal access to healthcare. Disaster preparedness should therefore extend beyond trauma care and include continuity of cardiovascular medications, preservation of emergency referral pathways, capacity to manage sudden increases in cardiovascular demand, and structured follow-up after the acute phase. This article reviews these cardiovascular consequences and discusses their implications for disaster preparedness in Colombia.

## 1 Introduction

The recent series of earthquakes across Latin America, including the most recent event in Colombia, has once again drawn attention to the consequences that extend well beyond the immediate economic, structural, and social toll. Although traumatic injuries and early mortality dominate the initial response, the health effects of earthquakes may persist long after the acute phase has passed. Cardiovascular disease is particularly relevant in this setting, as acute cardiovascular events can increase in the days following an earthquake, when disruption of healthcare, medications, and daily routines may contribute to a more sustained deterioration in cardiovascular risk (1,2,3).

This is particularly relevant for Colombia, where seismic events can affect territories with markedly different levels of healthcare access and response capacity, from geographically isolated areas such as Chocó to the more densely populated ones of the Coffee Region, southern Antioquia, and northern Valle del Cauca. Recognizing this broader cardiovascular burden is essential if disaster preparedness is to extend beyond trauma care and address both the immediate and longer-term needs of affected populations.

## 2 Cardiovascular Consequences After Earthquakes

Cardiovascular events appear to peak around three days after an earthquake, but the consequences are not confined to this acute window. Several studies report increases in all-cause mortality of up to 2% among affected populations, alongside substantial rises in cardiovascular mortality, with some studies documenting increases of up to 39%, largely driven by myocardial infarction and stroke (4,5,6). Beyond these acute events, earthquakes may also destabilize chronic cardiovascular and metabolic conditions. Glycated hemoglobin levels, for instance, tend to rise among patients with diabetes (5). Follow-up data from previous earthquakes suggest that these health consequences persist well beyond the acute phase, with increases in blood pressure, body weight, and dyslipidemia documented during the first year after the disaster (8).

Several mechanisms may explain this cardiovascular burden. Acute stress affects cardiac electrophysiology, increasing susceptibility to arrhythmias. Heightened autonomic activity may promote vascular changes, platelet activation, and ischemic alterations, which can in turn affect cardiac conduction (8). Beyond these acute pathways, the cardiovascular impact may be sustained by changes in traditional risk factors and lifestyle after the disaster. Disrupted sleep, reduced physical activity, psychological stress, and sustained sympathetic activation may contribute to worsening obesity, hypertension, diabetes, and dyslipidemia (5,6,8,9). These effects may be further amplified by displacement, medication interruption, changes in diet and daily routines, and prolonged disruption of healthcare.

## 3 Why Colombia Requires Particular Attention

In the Colombian context, the cardiovascular consequences of earthquakes are particularly relevant because major seismic events may simultaneously affect communities with very different baseline cardiovascular risk profiles and capacities to access timely care. These effects are likely to be greatest in vulnerable and resource-limited populations, where access to healthcare, medications, and essential supplies may already be constrained (3,7).

The earthquake of August 11 illustrates this concern. With its epicenter in Chocó, the event occurred in a region characterized by geographic isolation and important barriers to healthcare access, while its effects extended toward areas of the Coffee Region and western Colombia. This setting highlights how the cardiovascular consequences of an earthquake may vary substantially across affected populations. In more isolated communities, medication interruption, transportation difficulties, and delayed access to emergency or specialized cardiovascular care may become particularly important, whereas larger urban centers may face a sudden increase in demand for healthcare services.

This geographic heterogeneity is therefore not merely a logistical consideration. It may directly influence the ability of patients with acute myocardial infarction, stroke, arrhythmias, heart failure, or other cardiovascular conditions to obtain timely diagnosis and treatment. At the same time, disruption of routine care may destabilize patients with chronic cardiovascular disease who depend on uninterrupted access to medications, monitoring, and specialist follow-up.

## 4 Implications for Cardiovascular Preparedness

Earthquakes also place substantial pressure on healthcare systems, particularly cardiovascular services. Registries have reported increases of up to 41% in hospital admissions for cardiac conditions during the first week after an earthquake, including major adverse cardiovascular events and acute decompensation of chronic conditions such as heart failure (5,6,8). This surge in demand occurs precisely when infrastructure and healthcare resources may be most compromised, potentially delaying timely care.

Routine follow-up, medication delivery, scheduled diagnostic testing, and cardiovascular procedures may also be interrupted in the aftermath of a disaster (5,6,8). For Colombia, this represents a particular challenge because the impact may extend beyond the area surrounding the epicenter. Disruption of transportation and referral pathways in Chocó and neighboring territories could increase pressure on cardiovascular services in regional referral centers across the Coffee Region, Antioquia, and Valle del Cauca.

The cardiovascular impact of an earthquake should therefore be considered not only in terms of events occurring among directly affected populations, but also in terms of the capacity of the broader regional healthcare network to absorb a sudden increase in demand. Disaster planning should anticipate both immediate cardiovascular emergencies and the longer-term consequences of interrupted chronic disease management. These pathways, cardiovascular consequences, regional vulnerabilities, and preparedness priorities are summarized in Figure 1.

**Figure 1:**
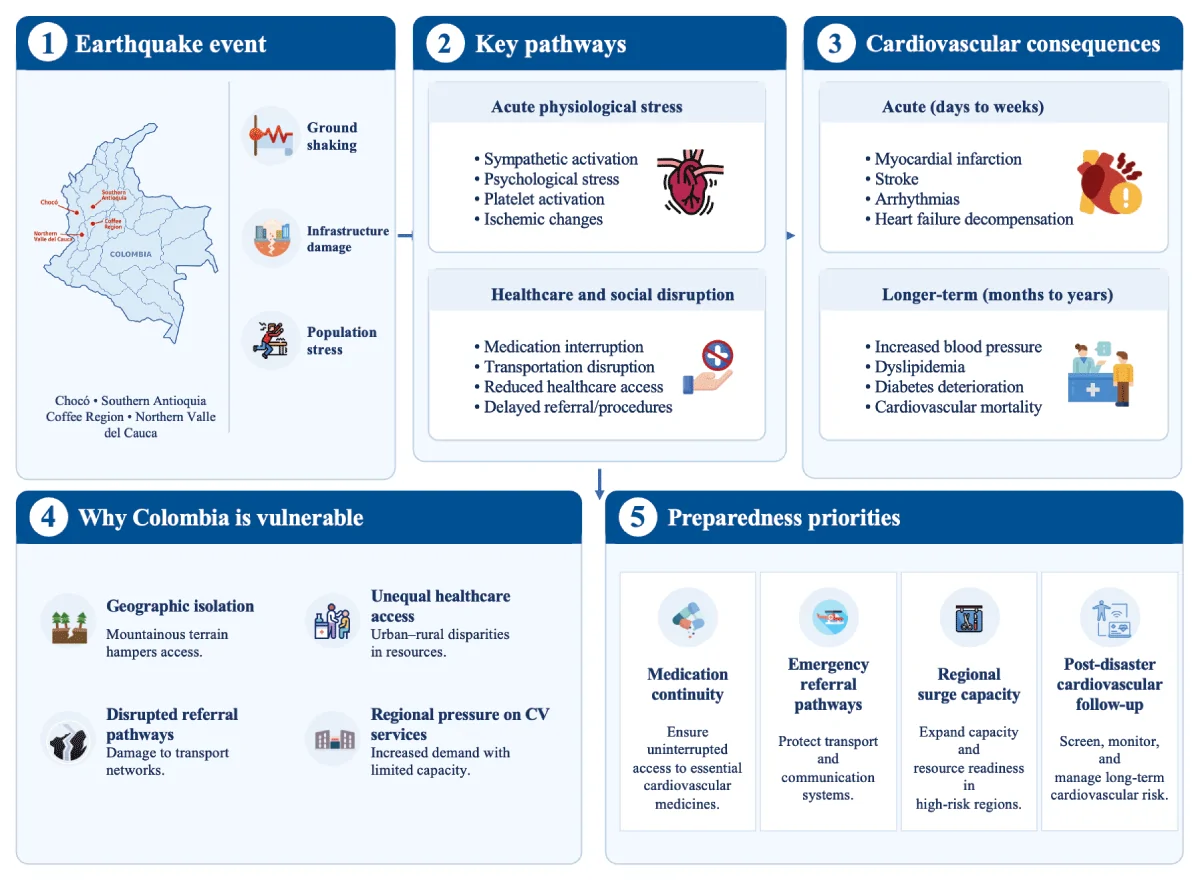
From seismic shock to cardiovascular burden: implications for disaster preparedness in Colombia. The figure illustrates the pathways through which earthquakes may affect cardiovascular health, from the initial seismic event and its associated physiological and healthcare-system disruptions to acute and longer-term cardiovascular consequences. In Colombia, geographic isolation, inequalities in healthcare access, disruption of referral pathways, and regional pressure on cardiovascular services may amplify these effects. Key preparedness priorities include continuity of essential cardiovascular medications, preservation of emergency referral pathways, adequate regional surge capacity, and structured post-disaster cardiovascular follow-up.

## 5 Discussion

Healthcare systems and cardiovascular professionals should recognize that cardiovascular disease burden may increase substantially after an earthquake and that preparedness cannot stop at trauma management and acute casualty care. Disaster-response strategies should anticipate not only an increase in acute cardiovascular events, but also the destabilization of chronic cardiovascular disease in the weeks and months that follow.

In Colombia, preparedness should account for the geographic and healthcare heterogeneity of potentially affected regions, particularly areas such as Chocó, the Coffee Region, southern Antioquia, and northern Valle del Cauca. Priorities should include continuity of essential cardiovascular medications, preservation of referral pathways for acute cardiovascular emergencies, adequate capacity to manage sudden increases in demand, and structured surveillance and follow-up of cardiovascular risk factors after the disaster (1,3,5,6,8).

Integrating these measures into disaster-response strategies may help reduce a cardiovascular burden that begins in the immediate aftermath of an earthquake but can persist long after the ground stops shaking.

## 6 Conclusions

Cardiovascular diseases are among the leading causes of mortality worldwide, and earthquakes may further increase their burden by triggering acute cardiovascular events and disrupting the care of patients with chronic conditions. Beyond the immediate impact of trauma, the cardiovascular consequences of a seismic event may persist for weeks or months, particularly in vulnerable populations with limited access to healthcare. In Colombia, geographic isolation, interruption of medication supply, and disruption of referral pathways may further amplify this risk. Disaster preparedness should therefore recognize cardiovascular care as an essential component of the response, ensuring continuity of treatment, timely access to emergency cardiovascular services, and appropriate follow-up after the acute phase. Incorporating these measures into disaster-response strategies may help reduce preventable cardiovascular morbidity and mortality and strengthen health-system resilience in future seismic events.

## Data Availability

No original data were generated or analyzed for this Viewpoint article. All information discussed was obtained from publicly available sources cited in the manuscript.
